# Downregulation of *Orco* and *5-HTT* Alters Nestmate Discrimination in the Subterranean Termite *Odontotermes formosanus* (Shiraki)

**DOI:** 10.3389/fphys.2019.00714

**Published:** 2019-06-11

**Authors:** Pengdong Sun, Shuxin Yu, Austin Merchant, Chaoliang Lei, Xuguo Zhou, Qiuying Huang

**Affiliations:** ^1^Hubei Insect Resources Utilization and Sustainable Pest Management Key Laboratory, College of Plant Science and Technology, Huazhong Agricultural University, Wuhan, China; ^2^Department of Entomology, University of Kentucky, Lexington, KY, United States

**Keywords:** nestmate discrimination, termites, chemosensation, neurotransmission, *in vivo* RNAi

## Abstract

Nestmate discrimination allows social insects to recognize nestmates from non-nestmates using colony-specific chemosensory cues, which typically evoke aggressive behavior toward non-nestmates. Functional analysis of genes associated with nestmate discrimination has been primarily focused on inter-colonial discrimination in Hymenopterans, and parallel studies in termites, however, are grossly lacking. To fill this gap, we investigated the role of two genes, *Orco* and *5-HTT*, associated with chemosensation and neurotransmission respectively, in nestmate discrimination in a highly eusocial subterranean termite, *Odontotermes formosanus* (Shiraki). We hypothesized that knocking down of these genes will compromise the nestmate recognition and lead to the antagonistic behavior. To test this hypothesis, we carried out (1) an *in vivo* RNAi to suppress the expression of *Orco* and *5-HTT*, respectively, (2) a validation study to examine the knockdown efficiency, and finally, (3) a behavioral assay to document the phenotypic impacts/behavioral consequences. As expected, the suppression of either of these two genes elevated stress level (e.g., vibrations and retreats), and led to aggressive behaviors (e.g., biting) in *O. formosanus* workers toward their nestmates, suggesting both *Orco* and *5-HTT* can modulate nestmate discrimination in termites. This research links chemosensation and neurotransmission with nestmate discrimination at the genetic basis, and lays the foundation for functional analyses of nestmate discrimination in termites.

## Introduction

Nestmate discrimination is the ability of social insects to recognize colony members using colony-specific recognition cues (Wilson, 1971). Depending on the species, recognition cues can be derived from a variety of biotic and abiotic sources (Hölldobler and Wilson, 1977). The process of nestmate discrimination involves two steps: recognition, where individuals that encounter one another assess whether the other is a nestmate on the basis of their recognition cues (Mateo, 2010); and action, where they adjust their behavior according to the chemical information perceived during recognition (Reeve, 1989; Baracchi et al., 2013). In the case of nestmates, individuals usually exhibit altruistic behaviors; otherwise they may exhibit stressful or aggressive behaviors (Hölldobler and Michener, 1980). Nestmate discrimination plays a crucial role in maintaining the stability of eusocial societies, ensuring that altruistic behaviors are directed only toward related individuals (Wilson, 1971).

Recognition and communication in social insects rely primarily on the olfactory system (Nehring and Steiger, 2018), which uses olfactory receptors (ORs) to convert olfactory cues into neural signals, which then elicit neuroendocrinological outputs and corresponding behavioral responses (Touhara and Vosshall, 2009). Eusocial insects must effectively discriminate recognition cues, and this capacity is reflected at the genetic level: the numbers of olfactory and gustatory receptor genes in the genomes of social insects are significantly greater than those in non-social insects (Terrapon et al., 2014; Harrison et al., 2018). The olfactory receptor co-receptor (Orco) is highly conserved across insect species (Nakagawa et al., 2012; Zhou et al., 2012), and is considered to form a tetramer which is arranged around specific pore and bound together by a small cytoplasmic anchor domain (Butterwick et al., 2018). Orco can form Orco-OR heterotetramers with all insect tuning ORs, the minimal sequence conservation among which maps largely to the pore and anchor domain (Butterwick et al., 2018). In solitary insects, the disruption of *Orco* can cause dramatic reductions in olfactory sensitivity, abolishing the behavioral, and electrophysiological responses to a number of general odorants (DeGennaro et al., 2013; Yang et al., 2016). In social insects, *Orco* mutant ants display a lack of social interactions, abnormal social behaviors, and reduced fitness (Trible et al., 2017; Yan et al., 2017). However, whether disruption of *Orco* expression can alter the process of nestmate discrimination is still unclear.

When individuals of social insects encounter non-nestmates, one common behavioral response is aggression (Liebert and Starks, 2004). Animal aggression may be affected and shaped by a large number of factors (Archer, 1988). In particular, serotonin (5-HT) has been implicated to be positively associated with aggression in a wide range of insect species, from solitary insects such as fruit flies (*Drosophila melanogaster*) and stalk-eyed flies (*Teleopsis dalmanni*) (Dierick and Greenspan, 2007; Bubak et al., 2014b) to social insects such as ants (*Formica rufa* and *Tetramorium caespitum*) (Kostowski and Tarchalska, 1972; Bubak et al., 2016). Moreover, the 5-HT levels of individuals that serve a defensive role in the colony are higher than those of their nestmates (Bubak et al., 2016; Ishikawa et al., 2016; Ohkawara and Aonuma, 2016). The serotonin transporter (5-HTT) is a key regulator of central serotonergic activity and can reuptake 5-HT from the synaptic cleft to the presynaptic neuron, thereby terminating its action at the synapse (Owens and Nemeroff, 1998). Mammal species show reduced aggression levels after treatment with 5-HT reuptake inhibitors (Miczek and Fish, 2005; Chichinadze et al., 2011), and knockdown of the *5-HTT* gene resulted in a reduction of aggression and home cage activity in mice (Holmes et al., 2002, 2003). However, the role of *5-HTT* in insects, specifically in the process of nestmate discrimination by social insects, has rarely been documented.

In the past few years, screening and functional analyses of genes associated with inter-colonial discrimination have focused primarily on Hymenoptera (Kravitz and Huber, 2003; Li-Byarlay et al., 2014; Toth et al., 2014; Chandrasekaran et al., 2015), and parallel studies in termites, however, are grossly lacking. To fill this gap, in this study, we investigated the role of two genes, *Orco* and *5-HTT*, associated with chemosensation and neurotransmission respectively, in nestmate discrimination in a highly eusocial subterranean termite, *Odontotermes formosanus* (Shiraki). Workers make up the majority of *O. formosanus* colonies and are responsible for foraging and colony maitantenance, i.e., frequently interacting with predators, competitors, and other biological stressors (Huang et al., 2006, 2007). Here, we hypothesized that knocking down of these genes will compromise the nestmate recognition and lead to the antagonistic behavior. To test this hypothesis, we carried out (1) an *in vivo* RNAi to suppress the expression of *Orco* and *5-HTT*, respectively, (2) a validation study to examine the knockdown efficiency, and finally, (3) a behavioral assay to document the phenotypic impacts/behavioral consequences.

## Materials and Methods

### Odontotermes Formosanus Colony Maintenance

Colonies of *O. formosanus* were collected from Wuhan city in Hubei province, China. A total of 16 colonies were used in this study (Table S1). Termites were maintained in sealed plastic containers in complete darkness (L:D = 0:24), at 25 ± 1°C and 75 ± 1% RH. All colonies were maintained under laboratory conditions without soil and with moist filter paper for 1 d before the subsequent experiments. After that, whole body samples of workers from 3 colonies were collected and stored at −80°C for tissue-specific gene expression analysis, and workers from the remaining 13 colonies were used for the RNAi experiments.

### Molecular Cloning

Total RNA from the whole bodies of 10 *O. formosanus* workers were extracted using TRIzol reagent (TaKaRa) according to the manufacturer's protocol, which was then treated with DNase I (TaKaRa) to remove genomic DNA. RNA quality was calculated and checked using a NanoDrop 2000 spectrophotometer (Thermo). The SMARTer RACE cDNA Amplification Kit (Clontech) was used to obtain the full-length sequences of *Orco* and *5-HTT* by amplifying both the 5′ and 3′ cDNA ends. Gene-specific primers (GSPs) for 5′- and 3′-RACE of *Orco* and *5-HTT* were designed based on the partial sequences of Unigene 55916 and Unigene 49370 (Table S2), respectively, which were obtained from transcriptome data of *O. formosanus* worker heads (Huang et al., 2012b). The amplification reactions were carried out as follows: 98°C for 3 min; 40 cycles of 98°C for 10 s, 60°C (touchdown to 55°C) for 10 s and 72°C for 30 s; and one cycle at 72°C for 7 min. The PCR products were purified using Wizard SV Gel Purification Kit (Promega). Purified PCR products were cloned into the pMD18-T vector (TaKaRa) followed by transformation in Trans1-T1 Phage Resistant Chemically Competent Cell (Transgen Biotech) according to the manufacturer's protocol. The plasmids were isolated from bacteria, and sequenced in both directions by Tsingke Biological Technology (Wuhan).

### Phylogenetic Analysis

Protein prediction was performed using ExPASy (http://web.expasy.org/translate/). cDNA and amino acid sequence similarity searches were performed using BLAST (https://blast.ncbi.nlm.nih.gov/Blast.cgi). Signal peptide and domain organization were predicted with SMART (http://smart.embl-heidelberg.de/). Protein multiple alignment analyses were performed using MEGA 6.0 and GenDoc 2.0 software. Phylogenetic analyses were performed using MEGA 6.0 software (Tamura et al., 2013). Amino acid sequences obtained from the NCBI database (Table S3) were transformed to a FASTA formatted file and uploaded to MEGA 6.0 software. Followed performing protein alignments using the ClustalW (Thompson et al., 1994), the phylogenetic relationships of *Orco*/*5-HTT* and their homolog genes in other species were analyzed using the neighbor-joining (NJ) method, with 1,000 bootstrap replications. MEGA 6.0 software was used to generate graphic of the sequence alignment.

### Tissue-Specific Expression Profile

Antenna, leg, head, and abdomen-thorax tissues of *O. formosanus* workers were dissected on an ice-cold plate. Per replicate, antennae of 100 individuals, legs of 30 individuals, and heads and abdomen-thoraxes of 15 individuals were used. Extraction and quality identification of total RNA were performed as described in “Molecular cloning and sequencing of *Orco* and *5-HTT*.” Approximately 1 μg of RNA was converted to cDNA using the PrimeScript^TM^ RT Reagent Kit with gDNA Eraser (Perfect Real Time) (TaKaRa). The RT-qPCR assay was performed using the My IQ^TM^ Color Real-time PCR Detection System (Bio-Rad) with cDNA as the template. Relative expression levels of *Orco* and *5-HTT* among the four tissue types were calculated using the 2^−ΔΔ*Ct*^ method (Van Hiel et al., 2009) with *Ribosomal protein S18* (*RPS18*) and *glyceraldehyde-3-phosphate dehydrogenase* (*GAPDH*) as reference genes. Five and six biological replicates were set for RT-qPCR of *Orco* and *5-HTT*, respectively. The primers used for RT-qPCR are listed in Table S2.

### *In vivo* Dietary RNAi Experiment

In order to construct a plasmid expressing dsRNA, fragments of *Orco* and *5-HTT* were amplified by RT-PCR using specific primers (Table S2), which were designed based on the full-length sequences of *Orco* and *5-HTT*. The restriction enzyme cutting sites of KpnI (Fermentas) and EcoRI (Fermentas) were added to the 5′ ends of the primers. PCR products were cloned to the L4440 plasmid, which has two T7 promoters in inverted orientation flanking the multiple cloning sites. The recombinant L4440-*Orco* and L4440-*5-HTT* plasmids were transformed to an HT115 (DE3) competent cell. Single colonies of HT115 (DE3) were shake-cultured in LB medium supplemented with 75 mg/mL ampicillin and 12.5 mg/mL tetracycline at 37°C overnight. The culture was diluted to 100-fold in 800 ml LB medium supplemented with 75 mg/mL ampicillin and 12.5 mg/mL tetracycline and shake-cultured at 37°C to OD600 = 0.5. Synthesis of dsRNA was induced by 0.4 mM IPTG, and then the bacteria were further incubated for 4 h at 37°C. The dsRNA of *Orco* and *5-HTT* were purified following the method described by Timmons et al. (2001). The quality of dsRNA was calculated and checked using a NanoDrop 2000 spectrophotometer (Thermo).

The dietary RNAi experiment was carried out by placing groups of 30 *O. formosanus* workers into 55 mm diameter petri dishes which were covered with treated filter paper. For both ds*Orco* and ds*5-HTT* feeding behavioral assays, the experimental design included one treatment [workers were fed using filter paper treated with 40 μL ds*Orco*/ds*5-HTT* (1.5 μg/μL) and Nile blue (0.5% w/v)], and one control [workers were fed using filter paper treated with 40 μL dsRNA for green fluorescent protein (ds*GFP*, 1.5 μg/μL) and Nile blue (0.5% w/v)] group. The workers used in dietary RNAi experiment were maintained in complete darkness (L:D = 0:24), at 25 ± 1°C and 75 ± 1% RH. The *O. formosanus* workers were maintained for 24 h, and then workers whose guts were colored by Nile blue were used in the gene silencing validation and behavioral assays.

### qRT-PCR Validation Study

After the dietary RNAi experiment, whole bodies of 5 *O. formosanus* workers were pooled and crushed in 1.5 mL centrifuge tubes with liquid nitrogen using sterilized grinding pestles. Total RNA extraction and cDNA synthesis of the samples were completed as described in “Molecular cloning and sequencing of *Orco* and *5-HTT*” and “Tissue-specific expression profiles of *Orco* and *5-HTT*.” The RT-qPCR assay was performed using the My IQ™ Color Real-time PCR Detection System (Bio-Rad) with cDNA as the template. Relative expression levels of *Orco* and *5-HTT* between workers fed with ds*Orco*/ds*5-HTT* and ds*GFP* were calculated using the 2^−ΔΔ*Ct*^ method (Van Hiel et al., 2009) with *RPS18* and *GAPDH* as reference genes. Gene silencing validation of *Orco* and *5-HTT* included three colonies with 8 and 7 replicates, respectively. The primers used for RT-qPCR are listed in Table S2.

### Behavioral Assay

After the dietary RNAi experiment, behavioral assays were carried out in a 35 mm diameter petri dish lined with moist filter papers. The behavioral assay for each gene included three groups, including (1) 1 target termite treated with ds*Orco*/ds*5-HTT* vs. 5 non-target termites treated with ds*GFP* (treatment group); (2) 1 target termite treated with ds*Orco*/ds*5-HTT* vs. 5 non-target termites treated with ds*Orco*/ds*5-HTT* (treatment group); and (3) 1 target termite treated with ds*GFP* vs. 5 non-target termites treated with ds*GFP* (control group). In each test group, the 5 non-target termites were first placed in the Petri dish to adapt to the environment for 10 min, and then the target termite, marked with red color on the pronotum, was added. Behavioral phenotypes of the termites in the Petri dish were recorded immediately with a digital camera (HDR-XR550, SONY). All Petri dishes were kept under laboratory conditions (25 ± 1°C, 75% ± 1% RH) and illuminated by a ceiling-mounted fluorescent lamp, which was necessary for the video-recording.

**Figure 1:**
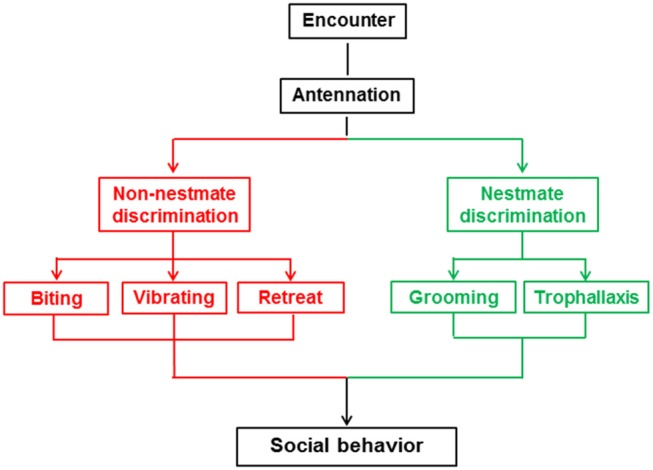
Social behaviors associated with nestmate discrimination in *O. formosanus*.

At the beginning of the behavioral assay and another three timepoints thereafter (30 min, 1 h, 2 h), we analyzed a 10 min section of video for the frequency and duration of five behaviors (biting, vibrating, retreat, grooming, trophallaxis) between the target and non-target workers, but did not analyze behaviors among the non-target termites. The five behaviors are shown in Figure 1 and Video S1 and described as follows: biting is an aggressive behavior in which one termite bites the body parts of another termite with the maxillae after the encounter (Tanner and Adler, 2009); vibrating is a distinctive behavior in which termites move repeatedly backwards and forwards after the encounter (Reinhard and Clément, 2002); retreat is a behavior in which termites move quickly in the opposite direction to avoid contact after the encounter (Martin et al., 2009); grooming is a behavior in which one termite licks the cuticle of a nestmate with its mouthpart after the encounter (Konrad et al., 2018); and trophallaxis is a behavior in which one termite transfers food or other fluids to a nestmate through mouth-to-mouth contact (Konrad et al., 2018). The *Orco* and *5-HTT* silencing behavioral assays included 10 colonies with 28 and 26 replicates, respectively.

## Results

### The Role of *Orco* in Nestmate Discrimination

#### Bioinformatic Analysis of *O. formosanus Orco*

**Figure 2:**
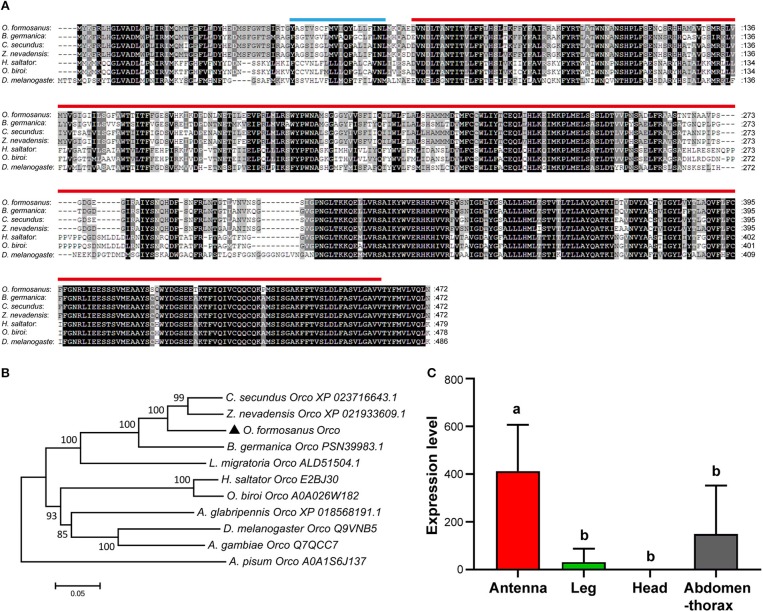
The structure, phylogeny and spatial expression of *O. formosanus Orco*. **(A)** Protein alignment of the *Orco* gene; the transmembrane domain is marked with a blue line, and the 7tm_6 domain is marked with a red line. **(B)** Phylogenetic tree of *Orco* in *O. formosanus* with homologs in other 10 species. **(C)** Expression patterns of *Orco* in antenna, leg, head, and abdomen-thorax tissue of workers (Tukey's HSD test, *P* < 0.05).

Based on the partial sequence of Unigene 55916 derived from transcriptome data of *O. formosanus* worker heads, a 1,737 bp nucleotide sequence representing the complete cDNA sequence of *Orco* was amplified. The cDNA of *Orco* included a 199 bp 5′ untranslated region (UTR) and a 119 bp 3′ UTR with a poly (A) tail. The open reading frame (ORF) of *Orco* was 1,419 bp and encoded a predicted protein of 472 amino acid (aa), with predicted molecular mass of 53.64 kDa and an isoelectric point (pI) of 6.89. SMART analysis showed that *Orco* contained a 20 aa transmembrane region and a 393 aa 7tm_6 domain (Figure 2A).

Multiple amino acid sequence alignments showed that *Orco* from *O. formosanus* shared the highest sequence identity with *Orco* genes from the termite *Zootermopsis nevadensis* (88%), the termite *Cryptotermes secundus* (87%), and the cockroach *Blattella germanica* (80%) (Figure 2A). Evolutionary analysis showed that *Orco* from *O. formosanus* was clustered with *Orco* from *Z. nevadensis, C. secundus, B. germanica*, and the locust *Locusta migratoria*, while *Orco* from two ant species (*Harpegnathos saltator* and *Ooceraea biroi*) was clustered in a different branch together with *Orco* from *D. melanogaster*, the longhorn beetle *Anoplophora glabripennis*, and the mosquito *Anopheles gambiae* (Figure 2B).

#### Tissue Distribution of *O. formosanus Orco*

The spatial distribution of gene expression may inform an initial understanding of the gene's function, therefore we performed qRT-PCR to profile the expression level of *Orco* in different tissue types (antenna, leg, head, and abdomen-thorax) of *O. formosanus*. The results showed that *Orco* exhibited the highest expression level in antennae, and there were no significant differences in the *Orco* expression among leg, head, and abdomen-thorax tissues (Figure 2C).

#### Behavioral Phenotype of *Orco* Knockdown

**Figure 3:**
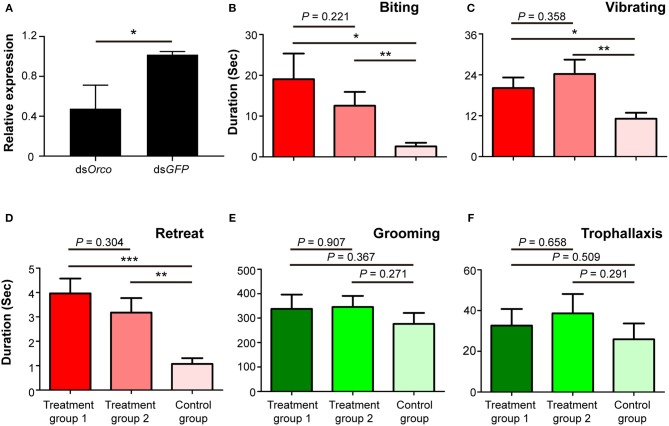
Phenotypic impact of *Orco* knockdown on nestmate discrimination. **(A)** The expression level of *Orco* after RNAi treatment. **(B)** Biting, **(C)** vibrating, **(D)** retreat, **(E)** grooming, and **(F)** trophallaxis behaviors modulated by silencing of *Orco*. The red columns represent social behaviors triggered by non-nestmate discrimination, including biting, vibrating, and retreat; the green columns represent social behaviors triggered by nestmate discrimination, including grooming and trophallaxis. Treatment group 1 consisted of 1 termite treated with ds*Orco* and 5 termites treated with ds*GFP*, treatment group 2 consisted of 1 termite treated with ds*Orco* and 5 termites treated with ds*Orco*, and the control group consisted of 1 termite treated with ds*GFP* and 5 additional termites treated with ds*GFP*. Data are shown as the means ± S.E.M. (paired *t*-test; *P < 0.05; **P < 0.01; ***P < 0.001).

To investigate the potential role of *Orco* in *O. formosanus* nestmate discrimination, RNAi-mediated silencing of *Orco* was performed in workers. The expression level of *Orco* was significantly suppressed in workers fed with ds*Orco* compared to workers fed with ds*GFP* 24 h after the dietary RNAi experiment (*df* = 7, *P* < 0.05; Figure 3A). This result indicates that RNAi effectively suppressed the expression of *Orco* in workers 24 h after the dietary RNAi experiment.

Behavioral assays were performed 24 h after the dietary RNAi experiment. The durations of stressful and aggressive behaviors between target and non-target workers were significantly higher in treatment groups 1 and 2 than in control groups. These behaviors were biting (treatment group 1: *t* = 2.553, *df* = 27, *P* < 0.05; treatment group 2: *t* = 2.931, *df* = 27, *P* < 0.01; Figure 3B), vibrating (treatment group 1: *t* = 2.757, *df* = 27, *P* < 0.05; treatment group 2: *t* = 3.151, *df* = 27, *P* < 0.01; Figure 3C), and retreat (treatment group 1: *t* = 4.649, *df* = 27, *P* < 0.001; treatment group 2: *t* = 3.483, *df* = 27, *P* < 0.01; Figure 3D). The duration of these behaviors between target and non-target workers in treatment groups 1 and 2 was not significantly different (Figures 3B–D). The durations of grooming and trophallaxis behaviors between target and non-target workers were not significantly different among the three groups (Figures 3E,F). The behavioral assays indicated that RNAi-mediated *Orco* silencing significantly altered social behaviors associated with non-nestmate discrimination (biting, vibrating, retreat) of workers in *O. formosanus*, but did not significantly influence social behaviors associated with nestmate discrimination (grooming and trophallaxis).

### The Role of 5-HTT in Nestmate Discrimination of *O. formosanus*

#### Bioinformatic Analysis of *O. formosanus* 5-HTT

**Figure 4:**
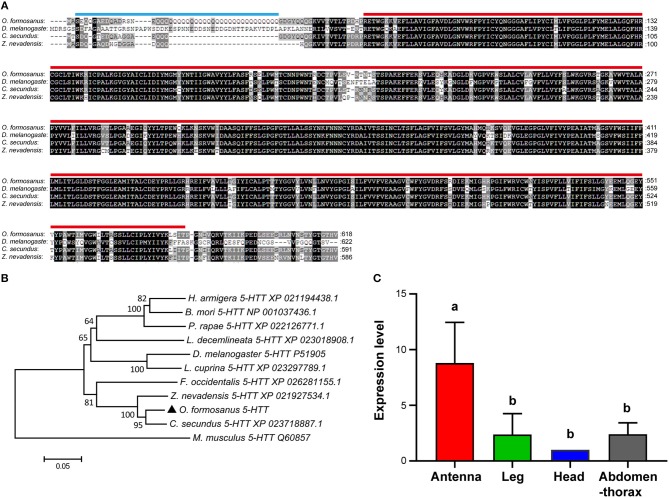
The structure, phylogeny, and spatial expression of *O. formosanus 5-HTT*. **(A)** Protein alignment of the *5-HTT* gene, the coiled coil domain is marked with a blue line, and the SNF domain is marked with a red line. **(B)** Phylogenetic tree of *5-HTT* in *O. formosanus* with homologs in 10 other species. **(C)** Expression patterns of *5-HTT* in antenna, leg, head and abdomen-thorax tissue of workers (Tukey's HSD test, *P* < 0.05).

Based on the partial sequence of Unigene 49370 derived from transcriptome data of *O. formosanus* worker heads, a 2,595 bp nucleotide sequence representing the complete cDNA sequence of *5-HTT* was amplified. The cDNA of *5-HTT* included a 728 bp 5′ UTR and a 343 bp 3′ UTR with a poly (A) tail. The ORF of *5-HTT* was 1,524 bp and encoded a predicted protein of 618 aa, with predicted molecular mass of 59.65 kDa and a pI of 6.71. SMART analysis showed that *5-HTT* contained a 44 aa Coiled coil domain and a 507 aa SNF domain (Figure 4A).

Multiple amino acid sequence alignments showed that *5-HTT* from *O. formosanus* shared the highest sequence identity with *5-HTT* genes from the termite *C. secundus* (94%), the termite *Z. nevadensis* (91%), and the thrips *Frankliniella occidentalis* (83%) (Figure 4A). Evolutionary analysis showed that *5-HTT* from *O. formosanus* was clustered with *5-HTT* from *Z. nevadensis, C. secundus*, and *F. occidentalis*. *5-HTT* from the mouse *Mus musculus* was clustered in a distinct branch apart from the *5-HTT* of insect species (Figure 4B).

#### Tissue Distribution of *O. formosanus* 5-HTT

Spatial analysis of *5-HTT* expression in different tissue types (antenna, leg, head, and abdomen-thorax) of *O. formosanus* showed that *5-HTT* exhibited the highest expression level in antennae, and there were no significant differences in the *5-HTT* expression among leg, head, and abdomen-thorax tissues (Figure 4C).

#### Behavioral Phenotype of 5-HTT Knockdown

**Figure 5:**
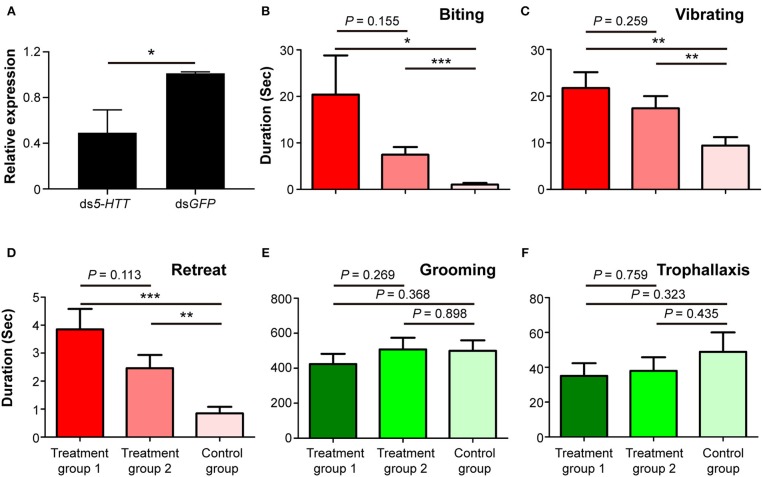
Phenotypic impact of *5-HTT* knockdown on nestmate discrimination. **(A)** The expression level of *5-HTT* after RNAi treatment. **(B)** Biting, **(C)** vibrating, **(D)** retreat, **(E)** grooming, and **(F)** trophallaxis behaviors modulated by silencing *5-HTT*. The red columns represent social behaviors triggered by non-nestmate discrimination, including biting, vibrating, and retreat; the green columns represent social behaviors triggered by nestmate discrimination, including grooming and trophallaxis. Treatment group 1 consisted of 1 termite treated with ds*5-HTT* and 5 termites treated with ds*GFP*, treatment group 2 consisted of 1 termite treated with ds*5-HTT* and 5 termites treated with ds*5-HTT*, and control group consisted of 1 termite treated with ds*GFP* and 5 additional termites treated with ds*GFP*. Data are shown as the means ± S.E.M. (paired *t*-test; *P < 0.05; **P < 0.01; ***P < 0.001).

The expression level of *5-HTT* was significantly suppressed in workers fed with ds*5-HTT* compared to workers fed with ds*GFP* 24 h after the dietary RNAi experiment (*df* = 6, *P* < 0.05; Figure 5A). This result indicates that RNAi effectively suppressed the expression of *5-HTT* in workers 24 h after the dietary RNAi experiment.

The results of behavioral assays following RNAi-mediated *5-HTT* silencing showed that the durations of stressful and aggressive behaviors between target and non-target workers were significantly higher in treatment groups 1 and 2 than in control groups. These behaviors were biting (treatment group 1: *t* = 2.303, *df* = 25, *P* < 0.05; treatment group 2: *t* = 4.122, *df* = 25, *P* < 0.001; Figure 5B), vibrating (treatment group 1: *t* = 3.509, *df* = 25, *P* < 0.01; treatment group 2: *t* = 3.369, *df* = 25, *P* < 0.01; Figure 5C), and retreat (treatment group 1: *t* = 4.316, *df* = 25, *P* < 0.001; treatment group 2: *t* = 3.139, *df* = 25, *P* < 0.01; Figure 5D). The duration of these behaviors between target and non-target workers in treatment groups 1 and 2 was not significantly different (Figures 5B–D). The durations of grooming and trophallaxis behaviors between target and non-target workers were not significantly different among the three groups (Figures 5E,F). The behavioral assays indicated that RNAi-mediated *5-HTT* silencing induced similar behavioral alterations in workers of *O. formosanus* as with *Orco* silencing, suggesting that the expression level of *5-HTT* can also affect the discrimination among *O. formosanus* workers from the same colony.

## Discussion

Nestmate discrimination of social insects is characterized by altruistic behaviors toward nestmates and stressful or aggressive behaviors toward non-nestmates (Huang et al., 2007, 2012a; Wenseleers et al., 2011; Konrad et al., 2018). Behaviors linked to nestmate discrimination vary across different social insect species (Reinhard and Clément, 2002). In termites, altruistic behaviors toward nestmates include grooming and trophallaxis (Nalepa, 2015), while stressful or aggressive behaviors toward non-nestmates mainly include biting, vibrating, and retreat behaviors (Reinhard and Clément, 2002; Martin et al., 2009; Tanner and Adler, 2009). Interactions between termites are direct reflections of the results of nestmate discrimination. In this study, we used *O. formosanus* to investigate the role of the *Orco* and *5-HTT* genes in nestmate discrimination through observation of the above-mentioned five social behaviors (biting, vibrating, retreat, grooming, and trophallaxis).

### Phenotypic Impact of *Orco* Knockdown

The Orco protein of *O. formosanus* contains a transmembrane region, which might aid in dimerization (Mukunda et al., 2014) and a 7tm_6 domain, which belongs to the insect chemoreceptor family (Goodstein et al., 2012; Benton, 2015). The results of our multiple amino acid sequence alignments and evolutionary analysis provide support for the idea that *Orco* is highly conserved across all insect species (Vosshall et al., 2000; Nakagawa et al., 2012; Zhou et al., 2012; Yan et al., 2017). In *O. formosanus, Orco* is predominantly expressed in the antennae. This expression pattern is similar to that of *Orco* orthologs in many other insect species, and further supports the idea that *Orco* plays critical roles in chemosensation (Malpel et al., 2008; Liu et al., 2013; Zhou et al., 2014; Franco et al., 2016).

The Orco protein is considered to form heterotetramer with all insect ORs for the purpose of chemosensation (Nakagawa et al., 2012; Zhou et al., 2012; Butterwick et al., 2018). Similar to observations in ant *Orco* mutants (Trible et al., 2017; Yan et al., 2017), our behavioral assays showed that the incidence of stressful behaviors (vibrating and retreat) between nestmates increased when *Orco* in one or both of them was knocked down, which was most likely due to a defect in sensing nestmate recognition cues (Yan et al., 2017). Furthermore, these results suggest that olfaction is important for accurate nestmate discrimination in termites, as in ants, although termites may have a limited ability to discriminate odors when compared to the eusocial Hymenoptera (Terrapon et al., 2014; Harrison et al., 2018). Knockdown of *Orco* increased the duration of biting behavior between nestmates, a behavioral phenotype that was more extreme than that observed in ant *Orco* mutants (Trible et al., 2017; Yan et al., 2017). These results might be due to a tolerance to non-nestmate odors, as in some ant or termite species that can peacefully coexist with heterospecific or non-nestmate conspecific competitors (Su and Haverty, 1991; Lenoir et al., 2001; Kaib et al., 2002; Boulay et al., 2004). *O. formosanus* individuals construct underground colonies in which foraging individuals are protected by mud shelters, and where a king-queen reproductive pair always resides (Huang et al., 2006). Colonies may be entrained to a relatively simple odor and show high sensitivity to dissimilar odors, so that the knockdown of *Orco* triggers aggressive behaviors (biting) among nestmates. Overall, these results suggest that the knockdown of *Orco* may cause a disruption of chemosensation in *O. formosanus*, which can degrade the ability to discriminate nestmates and non-nestmates, and ultimately trigger stressful (vibrating and retreat) and aggressive behaviors (biting) between nestmates.

### Phenotypic Impact of 5-HTT Knockdown

The 5-HTT protein of *O. formosanus* contains a coiled coil domain and an SNF domain, which belongs to the neurotransmitter transport system responsible for the removal of released neurotransmitters from the extracellular space (Attwell and Bouvier, 1992). Multiple amino acid sequence alignments and evolutionary analysis suggested that the *5-HTT* gene was highly conserved across different insect species but was distinct from that in mammal species. The antennae of insects contain an abundance of sensory neurons to process olfactory, gustatory, mechanosensory, hygrosensory, and thermosensory information (Watanabe et al., 2014; Versteven et al., 2017). 5-HT is secreted into the antennal hemolymph to modulate the responses of sensory neurons (Dolzer et al., 2001; Grosmaitre et al., 2001). Thus, the high expression of *5-HTT* in the antennae of *O. formosanus* might be responsible for the reuptake of 5-HT.

5-HT has been demonstrated to be positively associated with aggression in many insect species, such as fruit flies (*D. melanogaster*), stalk-eyed flies (*Teleopsis dalmanni*), and ants (*F. rufa* and *T. caespitum*) (Kostowski and Tarchalska, 1972; Dierick and Greenspan, 2007; Bubak et al., 2014b; Williams et al., 2014). However, this same association has not been observed in crickets (*Gryllus bimaculatus*) or ants (*Oecophylla smaragdina*) (Kamhi et al., 2015; Rillich and Stevenson, 2015). Our behavioral studies showed that incidence of stressful (vibrating and retreat) and aggressive behaviors (biting) among nestmates was significantly elevated following *5-HTT* knockdown, supporting the existence of species-specific relationships between 5-HT and aggression (Bubak et al., 2014a). These results were most likely due to the fact that *5-HTT* knockdown disrupts reuptake of 5-HT, which leads to an elevation of 5-HT in the synaptic cleft (Owens and Nemeroff, 1998), and therefore an increase in aggressive behavior. Moreover, 5-HT plays important role in the process of chemosensation by acting on peripheral neurons and brain centers. 5-HT can secret into the hemolymph to modulate the sensitivity of olfactory receptor neurons in insects' antennae (Dolzer et al., 2001; Grosmaitre et al., 2001). In the olfactory center of *Drosophila melanogaster* brain, 5-HT can enhance projection neuron responses by increasing the sensitivity of projection neuron (Dacks et al., 2009). In our study, there may have been slight differences in the odors of target and non-target workers which were separately maintained before the behavioral assays. Therefore, the enhanced sensitivity to different odors caused by *5-HTT* knockdown might be another interpretation of the elevated biting, vibrating, and retreat behaviors between target and non-target workers.

Collectively, we analyzed the primary sequence and spatial expression pattern of *Orco* and *5-HTT* in *O. formosanus* workers, which are respectively, associated with chemosensation and neurotransmission in insects. Our behavioral assays demonstrated that both *Orco* and *5-HTT* knockdown could trigger aggressive or stress responses (biting, vibrating, and retreat) among nestmates. These results suggest that downregulation of *Orco* and *5-HTT* expression can alter the nestmate discrimination of eusocial termites. Future studies on the neural circuit and nerve impulse transmission related to *Orco* and *5-HTT* is warranted to better understand the mechanism of nestmate discrimination in social animals.

## Data Availability

All datasets generated for this study are included in the manuscript and/or the Supplementary Files.

## Author Contributions

PS, XZ, and QH conceived and designed the experiments. PS and SY performed the experiments. PS, SY, and QH analyzed the data. PS, AM, CL, XZ, and QH drafted and revised the manuscript. All authors discussed the results and commented on the manuscript. All authors approved the final manuscript.

### Conflict of Interest Statement

The authors declare that the research was conducted in the absence of any commercial or financial relationships that could be construed as a potential conflict of interest.

## Supplementary Material

Video S1 Social interactions between one Odontotermes formosanus worker (marked in red) and five nestmates.

## Abbreviations

5-HT: serotonin
5-HTT: serotonin transporter
aa: amino acid
GFP: green fluorescent protein
GSPs: gene-specific primers
NJ: neighbor-joining
Orco: olfactory receptor co-receptor
ORF: open reading frame
ORs: olfactory receptors
pI: isoelectric point
UTR: untranslated region.
