# Supplementary material for: Downregulation of Orco and 5-HTT Alters Nestmate Discrimination in the Subterranean Termite Odontotermes formosanus (Shiraki)
Source: Front Physiol. 2019 Jun 11;10:714. doi: 10.3389/fphys.2019.00714 (PMC6579916; doi:10.3389/fphys.2019.00714)
Supplement: Supplementary file 1 [file Table_1.DOCX]

**Table S1 Number of *O. formosanus* colonies for each experiment.**

| **Experiments** | **Replicates** | **Number of colonies** |
| --- | --- | --- |
| Tissue-speciﬁc expression proﬁles of *Orco* | 5 | 3 |
| Tissue-speciﬁc expression proﬁles of *5-HTT* | 6 | 3 |
| *Orco* silencing validation by qRT-PCR | 8 | 3 |
| *5-HTT* silencing validation by qRT-PCR | 7 | 3 |
| Behavior assays after silencing of *Orco* | 28 | 10 |
| Behavior assays after silencing of *5-HTT* | 26 | 10 |
