# Supplementary material for: Downregulation of Orco and 5-HTT Alters Nestmate Discrimination in the Subterranean Termite Odontotermes formosanus (Shiraki)
Source: Front Physiol. 2019 Jun 11;10:714. doi: 10.3389/fphys.2019.00714 (PMC6579916; doi:10.3389/fphys.2019.00714)
Supplement: Supplementary file 2 [file Table_2.DOCX]

**Table S2 Primers used in this study.**

| **Gene Name** | **Orientation** | **(5′→3′) Primer Sequence** | **Purpose** |
| --- | --- | --- | --- |
| *Orco* | 3GSP | TGGACACTGTGGTGCCTAACTCCG | full-length amplification |
| *Orco* | 3NGSP | AGTGTACACATTGGCCCAGGTGTTTC | full-length amplification |
| *Orco* | 5GSP | TGGTGGTCCAGGCAAACCCGCT | full-length amplification |
| *Orco* | 5NGSP | ACTGCCATTGCATGGTGACGCGA | full-length amplification |
| *Orco* | Forward | AGCTCCTAGTACGGTCCGCCA | qRT-PCR |
|  | Reverse | TGTAGCAGCAGTGCCGACCC |  |
| *Orco* | Forward | TGGACACTGTGGTGCCTAAC | RNAi |
|  | Reverse | GATCACGGTGCAGGCATAGA |  |
| *5-HTT* | 3GSP | CTCAGCGTTCGCACTAACACTTCCACC | full-length amplification |
| *5-HTT* | 3NGSP | TGGGTCACAGCCTTGGCACCTTACG | full-length amplification |
| *5-HTT* | 5GSP | AGCGTCTTAGAGCAACACAAAGCAGATG | full-length amplification |
| *5-HTT* | 5NGSP | CCAGGGGCAGCAGAGGGAATCCAAT | full-length amplification |
| *5-HTT* | Forward | CGAGTATCCGAGGTTGTTAGG | qRT-PCR |
|  | Reverse | ACACCACGAAGAGTATTGAGATT |  |
| *5-HTT* | Forward | TCTCTCCTTGGTTACGAAGAGAT | RNAi |
|  | Reverse | TACTCTGACCTGTGTTACGTTAA |  |
| *RPS18* | Forward | ATGGCAAACCCCCGTCAGTA | internal references for qRT-PCR |
|  | Reverse | CATACCACGATGCGCACGAA |  |
| *GAPDH* | Forward | TCGTATTGGCCGTCTTGTGC | internal references for qRT-PCR |
|  | Reverse | AGCGACCATGGGTGGAATCAT |  |
