# Supplementary material for: Downregulation of Orco and 5-HTT Alters Nestmate Discrimination in the Subterranean Termite Odontotermes formosanus (Shiraki)
Source: Front Physiol. 2019 Jun 11;10:714. doi: 10.3389/fphys.2019.00714 (PMC6579916; doi:10.3389/fphys.2019.00714)
Supplement: Supplementary file 3 [file Table_3.DOCX]

**Table S3 List of species and corresponding accession numbers for sequences used in the phylogenetic analyses of *Orco* and *5-HTT*.**

| ***Orco*** |  | ***5-HTT*** |  |
| --- | --- | --- | --- |
| **Species** | **Accession number** | **Species** | **Accession number** |
| *Anoplophora glabripennis* | XP_018568191.1 | *Drosophila melanogaster* | P51905 |
| *Locusta migratoria* | ALD51504.1 | *Mus musculus* | Q60857 |
| *Blattella germanica* | PSN39983.1 | *Cryptotermes secundus* | XP_023718887.1 |
| *Cryptotermes secundus* | XP_023716643.1 | *Zootermopsis nevadensis* | XP_021927534.1 |
| *Zootermopsis nevadensis* | XP_021933609.1 | *Leptinotarsa decemlineata* | XP_008179499 |
| *Harpegnathos saltator* | E2BJ30 | *Adelphocoris suturalis* | XP_023018908.1 |
| *Ooceraea biroi* | A0A026W182 | *Frankliniella occidentalis* | XP_026281155.1 |
| *Drosophila melanogaster* | Q9VNB5 | *Helicoverpa armigera* | XP_021194438.1 |
| *Acyrthosiphon pisum* | A0A1S6J137 | *Bombyx mori* | NP_001037436.1 |
| *Anopheles gambiae* | Q7QCC7 | *Lucilia cuprina* | XP_023297789.1 |
|  |  | *Pieris rapae* | XP_022126771.1 |
